# Early childhood development under son preference in Vietnam: The role of child gender and birth order

**DOI:** 10.1371/journal.pgph.0006983

**Published:** 2026-08-27

**Authors:** Truc Thi Thanh Nguyen, Wiraporn Pothisiri

**Affiliations:** 1 Faculty of Public Health, University of Medicine and Pharmacy at Ho Chi Minh City, Ho Chi Minh City, Vietnam; 2 College of Population Studies, Chulalongkorn University, Bangkok, Thailand; International Centre for Diarrhoeal Disease Research, Bangladesh (icddr, b), BANGLADESH

## Abstract

Vietnam has experienced more than two decades of elevated prenatal sex selection due to persistent son preference, yet little is known about whether gender disparities emerge among surviving children in early childhood. This study examines gender differences in early childhood development (ECD) among Vietnamese children aged 24–59 months and investigates whether birth order and family context – key markers of son preference – moderate these differences. Using nationally representative data from the 2020–2021 Vietnam Multiple Indicator Cluster Survey, ECD was assessed across health, learning, and psychosocial domains – using the UNICEF framework. Multivariate logistic regression models were estimated and adjusted for child, mother, home, and household characteristics, including interaction terms to test moderating effects. Among 2,354 children, 21.3% were developmentally off track. Overall, no gender differences in ECD were observed. However, gender disparities emerged when birth order was considered: firstborn daughters had the highest developmental probabilities, whereas firstborn sons had the lowest. These patterns likely reflect a combination of selection into less son-preferring families, differences in biological susceptibility, and gendered parental investment shaped by cultural expectations. Other significant predictors of positive ECD included the absence of functional difficulties, higher maternal education, greater household wealth, access to children’s books, and greater family engagement in stimulating activities. Vietnam provides a unique context in which strong prenatal son preference coexists with largely neutral gender outcomes in early childhood. Nonetheless, birth-order-specific gender patterns highlight the importance of family dynamics in shaping developmental opportunities. Continued monitoring is needed to assess whether gender disparities may emerge as fertility further declines and family structure evolves.

## Introduction

Early childhood development (ECD) plays a critical role in shaping future outcomes, from early learning opportunities and academic achievement to long-term economic prospects and social competencies [1,2]. Life course theory links early-life development in cognitive, emotional, and social skills to later outcomes, emphasising how age shapes personal experiences and transitions within broader cultural and social norms and historical contexts [3]. Investments in the early years – through quality education, nutrition, healthcare, nurturing environments, security, and safety – yield significant returns by improving life chances and reducing violence, crime, and social inequality in adulthood [4].

These efforts align closely with the United Nations Sustainable Development Goals (SDGs), particularly SDG 4 (Quality Education), SDG 3 (Good Health and Well-Being), and SDG 10 (Reduced Inequalities), highlighting the global urgency of prioritising ECD in public health policy and practice [5]. In response, the World Health Organization (WHO) introduced the Nurturing Care Framework for ECD as an international standard to help countries achieve the SDGs [4]. Similarly, the United Nations Children’s Fund (UNICEF) emphasises the importance of a caring and supportive home environment in promoting child development [6].

A comprehensive understanding of the determinants of ECD within a given country context is essential for supporting child development. Bronfenbrenner’s ecological systems theory emphasises the complexity of environmental influences on children’s experiences and growth. However, caution is required when making broad assumptions about individuals based solely on their environmental contexts, as each child has the capacity to thrive despite challenging circumstances. Moreover, development is not static; it evolves and is influenced by various life events and transitions. Therefore, interventions to enhance child development are always timely and worthwhile, regardless of age or circumstance [7].

The ecological model of family influences further emphasises the reciprocal relationship between child development and the family environment. Families provide children with food, shelter, and stability, and play a crucial role in their emotional development. Children learn about social and emotional connections primarily at home. Family life is shaped by past experiences, leading to predictable routines and the transmission of values across generations, with parents serving as the most influential figures. At the same time, children are not passive recipients; their characteristics, such as gender and temperament, also shape how they interact with and fit into their families [8].

Furthermore, resource dilution theory states that when more children are added to the family, economic and parental resources eventually diminish, resulting in better outcomes for earlier-born children due to increased resource availability. These variations are caused by postnatal factors, such as the home environment, rather than by genetic or in utero differences [9]. Importantly, resource dilution is not gender neutral but is affected by a country’s level of gender inequality. In gender-inequality communities, parents are more likely to prioritise sons’ schooling over daughters’, exacerbating the detrimental impact of having siblings on women’s educational outcomes [10]. Empirical studies have also found that son preference culture is associated with various negative developmental outcomes for girls worldwide, including lower-than-expected survival rates in 15 countries [11] and lower height-for-age and weight-for-age z-scores in 66 developing countries [12]. Girls’ discrimination in immunisation coverage, treatment-seeking [13], nutrition status [14], and breastfeeding has been documented in India [15], as well as less nutritious food and poorer healthcare in China [16,17]. Over time, these disparities contribute to persistent gender gaps in education, employment, and health that extend into adulthood and old age [18,19].

Son preference is a cultural and social phenomenon prevalent in many societies, particularly those with Confucian heritage, such as China and Vietnam [20,21], where sons are valued more than daughters for various reasons, including the continuation of the family line, participation in religious and cultural rituals, and the provision of financial and emotional support to parents in their old age [22]. According to the value of children theory, this gender preference influences parental practices, leading to gender bias in nurturing in early childhood and resource investments later in life in children of the more valued gender [23].

Vietnam currently ranks fourth in the world for sex ratio at birth (SRB), with 110.6 in 2024 [24], well above the normal range (104–107) [25] and above other Asian countries, including China (110.3). This imbalance is the most serious demographic challenge in Vietnam for over two decades, with SRB increasing from 107 in 1999–112 in 2021 [26], driven by patrilineal cultural norms [19], resulting in a high prevalence of missing female births during 2009–2019 [25]. Despite severe prenatal sex selection, Vietnam provides a unique context, with no evidence of excess female mortality [19]. Recent global estimates confirmed the absence of excess under-five female deaths in Vietnam in both 1990 and 2021, in contrast to findings from other son-preference countries such as China and India [27].

Empirical evidence on the effects of son preference on ECD in Vietnam remains limited. Most existing studies have focused on single indicators such as malnutrition [28], immunisation [29], or breastfeeding [30], often highlighting socioeconomic inequalities in these outcomes but rarely examining them from a gender perspective. Furthermore, birth order and the gender composition of siblings also significantly influence gender preferences in Vietnam [19], possibly leading to differences in parental treatment in early childhood. These two factors have been considered mediators in the relationship between gender and child development, as noted in prior research [13,14]. Yet no study has investigated how these family-level factors jointly influence gender-specific child development in the Vietnamese context. Understanding these patterns is essential for identifying vulnerable groups and informing public health policies aimed at reducing inequalities in ECD.

Given this context, the study addresses gaps in ECD research by examining gender differences through the lens of family dynamics and cultural norms, marking the first such investigation in Vietnam, where son preference remains prominent [19,25,31]. While much attention has focused on the prenatal consequences of son preference, less is known about the postnatal development of girls who are born despite this cultural bias. In other words, does gender preference at birth persist after childbirth in ways that influence children’s developmental trajectories? The following research questions guide this study: (1) What are the developmental patterns among Vietnamese children aged 24–59 months, and do these patterns differ by gender? To what extent might any observed differences reflect underlying cultural preferences for sons? (2) How are family-level structural characteristics – specifically birth order and gender composition of siblings – and broader contextual factors associated with ECD outcomes?

## Materials and methods

### Data and sample

This study utilised data from the nationally representative cross-sectional Vietnam MICS 2020–2021 (MICS6), which is publicly available on the official UNICEF website. UNICEF developed the Global MICS Program in the mid-1990s to monitor the situation of children and women worldwide and to provide internationally comparable data [32]. The General Statistics Office of Vietnam (GSO) conducted the fieldwork between 18 November 2020 and 3 February 2021, through face-to-face interviews, with technical and financial support from UNICEF and the United Nations Population Fund (UNFPA). The survey employed a two-stage stratified cluster sampling design based on the 2019 Vietnam Census. Enumeration areas were selected systematically with probability proportional to size, followed by a random selection of 20 households per cluster. Further details on MICS6 are available elsewhere [33].

For this study, the sample comprised 2,473 children aged 24–59 months whose biological mothers had completed the prior interviews. Cases with missing or invalid data on key analytical variables were excluded, resulting in the removal of 119 cases (4.8% of the initial sample). The final analytic sample included 2,354 children.

### Ethics statement

This study utilised secondary data from the Vietnam MICS6 2020–2021, which is publicly available on the UNICEF website. Since the dataset is anonymous and de-identified, this analysis did not require primary Institutional Review Board approval.

### Variable measurements

#### Outcome variable.

The primary outcome variable in this study was children’s ECD status, assessed among those aged 24–59 months using UNICEF’s Early Childhood Development Index 2030 (ECDI2030). Although the MICS6 dataset included the 20 ECDI2030 items, the composite index was not pre-constructed; therefore, we derived the ECD score following UNICEF’s recommended procedure [1].

Mothers were asked 20 questions covering their children’s behaviours, abilities, and knowledge across three developmental domains: health, learning, and psychosocial development. The full list of questions is available in Vietnam MICS6 [33]. Each response was recoded into a binary score (0 or 1) and summed, generating a total score ranging from 0 to 20.

The final outcome variable was a binary indicator of whether a child was developmentally “on track.” A child was classified as on track (coded 1) if their total score met the minimum age-specific threshold recommended by UNICEF: ≥ 7 (24–29 months), ≥ 9 (30–35 months), ≥ 11 (36–41 months), ≥ 13 (42–47 months), and ≥15 (48–59 months) [1]. Children scoring below the threshold were coded as “off track” (coded 0).

#### Key explanatory variables.

Child gender was the main explanatory variable, coded as 1 for females and 0 for males. In addition to the child’s own gender, we also constructed a measure of sibling gender composition. This variable was derived solely from co-resident siblings to reflect the gender environment within the household at the time of the interview. It was coded as 1 if the child had at least one older brother and 0 otherwise.

Birth order was also defined by the order of children currently living with their mothers at the time of the survey. Although women’s birth histories provided information on the birth order of all children ever born, including those who had died or were living elsewhere, we restricted the measure to co-resident children because the study aimed to examine how birth order and gender interact within the household context. Birth order was categorised into four groups: only child, first child, second child, and third child or higher.

Ethnicity referred to the household head’s ethnicity and was classified into two categories: Kinh and Hoa (majorities) and minorities. The six socio-economic regions included Red River Delta (Red River), Northern Midlands and Mountains (Northern Midlands), North Central and Central Coastal (North Central), Central Highlands, South East, and Mekong River Delta (Mekong River), according to Resolution 138/NQ-CP dated 25 October 2022 [34].

#### Covariates.

A range of child, mother, home environment, and household characteristics were included as covariates. The child’s age was measured in years and divided into three categories: 2, 3, and 4 years old. A child was coded 1 for having a functional difficulty if they had trouble in at least one domain: seeing, hearing, walking, fine motor skills, communication, learning, or playing, and 0 otherwise. The mother’s age at birth was calculated by subtracting the child’s age from the mother’s age in completed years at the time of the interview and categorised into three age groups: 12–24, 25–29, and 30–44 years old. Mother’s education referred to the highest level of education completed and the grade of school attended at the time of the interview, including primary and below (illiteracy to grade 5), lower secondary (grades 6–9), and upper secondary and above (grades 10–12, vocational high schools, college, and university). Marital status had three categories: currently married, formerly married, and never married. Marriage included individuals who were married or living with someone as if married.

Home environment variables captured various stimuli and resources. These included the number of books or picture books available to the child at the time of the interview, categorised as none, 1–9 books, or 10 or more books. Child’s engagement was defined as the number of stimulating activities – such as reading books, telling stories, singing songs, taking children outside, playing with them, naming, counting, or drawing things – that at least one household member engaged in with children in the past three days. This variable was divided into two groups: 0–4 activities and 5–6 activities.

Child’s discipline was assessed based on mothers’ responses to 11 predefined disciplinary practices used in the past month, with responses recorded as yes (1) or no (0). Following standard UNICEF guidelines, these practices are typically grouped into three categories: (i) non-violent discipline (e.g., explanation or redirection), (ii) psychological aggression (emotional violence), and (iii) physical punishment. These are conceptually distinct, and children in our sample may be exposed to multiple forms of discipline simultaneously or to none. *Violent discipline* is defined as exposure to either psychological aggression or physical punishment. For this analysis, the child’s discipline was classified into four distinct categories: (i) no discipline, (ii) only non-violent discipline, (iii) psychological aggression without physical punishment, and (iv) any physical punishment. For brevity, these categories are denoted in tables as *no discipline, non-violent discipline, psychological aggression, and physical punishment*, respectively.

Household characteristics included wealth, measured by the wealth index (coded as 1 for rich or richest and 0 for the rest), and residence (1 for urban and 0 for rural). Household size referred to the number of individuals in the household at the time of the interview (1 for 5–17 members and 0 for 2–4 members).

### Statistical analysis

All analyses were conducted using Stata 17. Sample weights provided in the MICS6 dataset were applied to account for the complex survey design and to produce nationally representative estimates. We began with descriptive analyses, presenting weighted frequencies and percentages. Differences in ECD status across child, maternal, home environment, and household characteristics were examined using chi-square tests.

To assess the associations between ECD and key explanatory variables, we used a series of sequential multivariate logistic regression models. Model 1 included the key predictors: child gender and birth order. Model 2 added child characteristics, followed by Model 3, which incorporated maternal characteristics, the home environment, and other household covariates. The final model (Model 4) added an interaction term between child gender and birth order [14]. Results were reported as odds ratios (ORs), with statistical significance set at *p* < .05 [35].

To explore potential moderating factors, we initially examined interaction terms for birth order, sibling gender composition, ethnicity, and region. These were selected based on prior empirical evidence that Vietnamese families may hold son preferences or mixed-gender preferences, and that such preferences may vary by ethnic group, region, and sibling composition [19,25,36–38]. Among the interaction terms, only the interaction between gender and birth order was statistically significant and retained in the final model (Model 4). To facilitate interpretation of the interaction term, we estimated average predicted probabilities of being developmentally on track for girls and boys across different birth-order groups, controlling for other covariates.

Several robustness checks were performed to ensure the stability of the analysis. These included (1) using a probit model (S1 Table), (2) re-estimating the model without survey weights (S2 Table), (3) excluding only children (S3 Table), and (4) excluding households with multiple siblings (S4 Table). The direction and significance of key variables were consistent across specifications.

Model diagnostics were conducted to assess fit and multicollinearity. The Hosmer–Lemeshow goodness-of-fit test indicated satisfactory model fit for all four models (*p* > .05) [39]. Model comparisons using Akaike Information Criterion (AIC) and the Bayesian Information Criterion (BIC) showed that Model 4 provided the best fit, with the lowest AIC and BIC values [40]. Variance inflation factor (VIF) values for all key explanatory variables and covariates were below 5 (mean VIF = 2.59), indicating no problematic multicollinearity [41].

## Results

### Characteristics of the study sample

Table 1 presents the characteristics of the 2,354 Vietnamese children aged 24–59 months included in the analysis and shows how the proportion of children who were developmentally on track varied across groups. Overall, 78.7% of children were classified as on track in 2020–2021, while 21.3% were off track. These figures differ slightly from the MICS6 report [33] due to additional data cleaning conducted for this study.

**Table 1:** Characteristics of the study sample by ECD, Vietnam, 2020–2021 (N = 2,354).

Characteristics		Early childhood development		χ^2^	*p-*value
	n (%)	On track	Off track		
Early childhood development					
Off track	525 (21.3)	–	–	–	–
On track	1935 (78.7)	–	–	–	–
Child’s gender					
Boy	1274 (51.8)	976 (76.6)	298 (23.4)	6.6	.057
Girl	1186 (48.2)	960 (80.9)	226 (19.1)		
Birth order					
First child	391 (15.9)	298 (76.1)	93 (23.9)		
Only child	543 (22.1)	444 (81.8)	99 (18.2)	15.0	.033
Second child	1201 (48.8)	961 (80.1)	240 (19.9)		
Third child+	325 (13.2)	233 (71.6)	92 (28.4)		
Gender composition of siblings					
Having an older brother	798 (32.4)	609 (76.4)	189 (23.7)	3.64	.136
No older brother	1662 (67.6)	1326 (79.8)	336 (20.2)		
Child’s age					
2 years	734 (29.8)	606 (82.5)	128 (17.5)		
3 years	836 (34.0)	664 (79.5)	172 (20.5)	14.2	.023
4 years	890 (36.2)	665 (74.8)	224 (25.2)		
Functional difficulty					
No	2432 (98.9)	1930 (79.4)	502 (20.6)	60.3	< .001
Yes	28 (1.1)	5 (17.5)	23 (82.5)		
Mother’s age at birth					
12–24	777 (31.6)	587 (75.5)	190 (24.5)		
25–29	904 (36.7)	746 (82.5)	158 (17.5)	12.7	.030
30–44	779 (31.7)	603 (77.4)	176 (22.6)		
Mother’s education					
Primary and below	247 (10.1)	146 (59.0)	101 (41.0)		
Lower secondary	711 (28.9)	542 (76.1)	169 (23.9)	74.3	< .001
Upper secondary+	1501 (61.0)	1248 (83.1)	253 (16.9)		
Marital status					
Currently married	2387 (97.0)	1885 (79.0)	502 (21.0)		
Formerly married	67 (2.8)	48 (71.7)	19 (28.3)	10.0	.020
Never married	6 (0.2)	2 (29.0)	4 (71.0)		
Providing books					
None	1449 (58.9)	1062 (73.3)	387 (26.7)		
1–9 books	623 (25.3)	517 (83.1)	106 (16.9)	68.1	< .001
10 + books	388 (15.8)	356 (91.7)	32 (8.3)		
Child’s engagement					
0–4 activities	1297 (52.7)	949 (73.1)	348 (26.8)	47.9	< .001
5–6 activities	1163 (47.3)	987 (84.8)	176 (15.1)		
Child’s discipline					
No discipline	131 (5.3)	84 (64.1)	47 (35.9)		
Non-violent discipline	536 (21.8)	422 (78.8)	114 (21.2)	23.3	.004
Psychological aggression	482 (19.6)	404 (83.8)	78 (16.2)		
Physical punishment	1311 (53.3)	1025 (78.2)	286 (21.8)		
Household wealth					
Others	1423 (57.9)	1031 (72.4)	392 (27.6)	74.9	< .001
Rich/richest	1036 (42.1)	904 (87.2)	132 (12.8)		
Household size					
2–4 members	1207 (49.1)	936 (77.6)	271 (22.4)	1.67	.351
5–17 members	1253 (50.9)	999 (79.8)	254 (20.3)		
Ethnicity					
Minorities	415 (16.9)	266 (64.1)	149 (35.9)	60.3	< .001
Kinh and Hoa	2045 (83.1)	1669 (81.6)	376 (18.4)		
Residence					
Rural	1642 (66.7)	1253 (76.3)	389 (23.7)	15.6	.003
Urban	818 (33.3)	682 (83.4)	136 (16.6)		
Region					
Red River Delta	621 (25.3)	539 (86.8)	82 (13.2)		
Northern Midlands	379 (15.4)	265 (70.0)	114 (30.0)		
North Central	543 (22.1)	419 (77.3)	54 (22.7)	49.1	< .001
Central Highlands	184 (7.5)	130 (70.9)	54 (29.1)		
South East	394 (16.0)	304 (77.1)	90 (22.9)		
Mekong River Delta	340 (13.8)	278 (81.9)	62 (18.1)		

Authors’ calculations based on Vietnam MICS 2020–2021. svy was used to account for the survey design. Weighted n and % are reported.

Boys represented 51.8% of the sample. The age distribution of children was relatively even across the three age groups, although the proportion of children who were on track declined with age, from 82.5% among 2-year-olds to 74.8% among 4-year-olds. In terms of birth order, nearly half of the children were second-born (48.8%), 22.1% were only children, 15.9% were firstborn with younger siblings, and 13.2% were third-born or later. Two-thirds of children had no older brother (67.6%). Functional difficulty was rare (1.1%) but strongly associated with being off track.

Maternal characteristics showed significant variations in ECD outcomes. Children born to mothers aged 25–29 had the highest proportion on track (82.5%), compared with 77.4% for mothers aged 30–44 and 75.5% for younger mothers aged 12–24. Maternal education showed a particularly strong gradient: only 59.0% of children whose mothers had primary education or below were on track, compared with 76.1% for lower secondary and 83.1% for upper secondary education or higher.

Providing books appears to have a positive impact on children’s development, as most children (91.7%) with 10 or more books were on track, compared to 73.3% of those with no books. However, more than half of households (58.9%) did not provide any books for their children. Nearly half of children (47.3%) were engaged with family members in 5–6 activities – reading books, telling stories, singing songs, taking children outside, playing with them, naming, counting, or drawing things – resulting in a higher on track proportion of 84.8%. Children exposed to psychological aggression without physical punishment accounted for 19.6% and were more likely to be on track (83.8%) than other groups of children.

Additionally, 42.1% of the children lived in the wealthiest families, 83% belonged to the Kinh and Hoa ethnic groups, and 66.7% resided in rural areas. The sample was distributed across six socio-economic regions of Vietnam. Children who lived in the wealthiest households, in urban areas, or belonged to the majority ethnicity had higher proportions of being developmentally on track (87.2%, 83.4%, and 81.6%, respectively), compared to those without these characteristics.

### Gender differences in early childhood development across birth order

Table 2 shows the results of four multivariate logistic regression models of ECD. Model 1, which included child gender and birth order, showed that gender did not significantly affect development status because the *p-*value was .048 and the confidence interval (CI) likely included 1. Birth order also showed an insignificant association with ECD (*p* > .05).

**Table 2:** Multivariate logistic regression models on gender differences in ECD among children aged 24–59 months (N = 2,354).

Variables	Likelihood of being on track for development			
	Model 1	Model 2	Model 3	Model 4
	OR (95% CI)	OR (95% CI)	OR (95% CI)	OR (95% CI)
Child’s gender (Ref. Male)				
Female	1.32 (1.003–1.72)*	1.31 (0.99–1.74)	1.33 (0.98–1.80)	3.34 (1.46–7.63)**
Birth order (Ref. First child)				
Only child	1.43 (0.93–2.21)	1.21 (0.77–1.91)	1.27 (0.76–2.11)	2.98 (1.47–6.04)**
Second child	1.26 (0.86–1.85)	1.21 (0.78–1.88)	1.14 (0.69–1.86)	1.58 (0.77–3.23)
Third child+	0.79 (0.50–1.25)	0.77 (0.45–1.34)	0.71 (0.37–1.34)	1.10 (0.47–2.43)
Interaction: Female × Birth order				
Female × Only child	–	–	–	0.14 (0.05–0.38)***
Female × Second child	–	–	–	0.45 (0.17–1.16)
Female × Third child+	–	–	–	0.38 (0.13–1.11)
Gender composition of siblings (Ref. Having an older brother)				
No older brother	–	1.17 (0.83–1.64)	1.05 (0.74–1.50)	1.04 (0.73–1.49)
Child’s age (Ref. 2 years)				
3 years	–	0.81 (0.57–1.16)	0.73 (0.50–1.05)	0.73 (0.51–1.05)
4 years	–	0.62 (0.44–0.86)**	0.52 (0.36–0.76)**	0.52 (0.36–0.76)**
Functional difficulty (Ref. No)				
Yes	–	0.05 (0.01–0.20)***	0.05 (0.01–0.24)***	0.05 (0.01–0.20)***
Mother’s age at birth (Ref. 12–24)				
25–29	–	–	1.19 (0.83–1.71)	1.20 (0.83–1.73)
30–44	–	–	1.04 (0.68–1.59)	1.03 (0.67–1.57)
Mother’s education (Ref. Primary and below)				
Lower secondary	–	–	1.63 (1.06–2.50)*	1.65 (1.08–2.54)*
Upper secondary+	–	–	1.53 (1.00–2.37)	1.58 (1.01–2.47)*
Marital status (Ref. Currently married)				
Formerly married	–	–	0.66 (0.32–1.37)	0.68 (0.31–1.46)
Never married	–	–	0.15 (0.02–1.00)	0.10 (0.01–0.70)*
Providing books (Ref. None)				
1–9 books	–	–	1.40 (0.94–2.10)	1.42 (0.97–2.09)
10 + books	–	–	2.40 (1.33–4.31)**	2.40 (1.34–4.29)**
Child’s engagement (Ref. 0–4 activities)				
5–6 activities	–	–	1.48 (1.09–2.01)*	1.48 (1.09–2.02)*
Child’s discipline (Ref. No discipline)				
Non-violent discipline	–	–	1.35 (0.78–2.33)	1.32 (0.75–2.31)
Psychological aggression	–	–	2.11 (1.14–3.90)*	2.05 (1.09–3.84)*
Physical punishment	–	–	1.47 (0.85–2.55)	1.38 (0.79–2.39)
Household wealth (Ref. Others)				
Rich/richest	–	–	1.53 (1.03–2.28)*	1.56 (1.04–2.33)*
Household size (Ref. 2–4 members)				
5–17 members	–	–	1.30 (0.96–1.75)	1.28 (0.95–1.98)
Ethnicity (Ref. Minorities)				
Kinh and Hoa	–	–	1.37 (0.95–1.97)	1.37 (0.94–1.98)
Residence (Ref. Rural)				
Urban	–	–	1.11 (0.76–1.60)	1.12 (0.77–1.63)
Region (Ref. Red River)				
Northern Midlands	–	–	0.77 (0.46–1.29)	0.76 (0.45–1.27)
North Central	–	–	0.64 (0.37–1.08)	0.62 (0.36–1.05)
Central Highlands	–	–	0.80 (0.44–1.46)	0.82 (0.45–1.51)
South East	–	–	0.56 (0.35–0.90)*	0.55 (0.34–0.89)*
Mekong River	–	–	1.08 (0.58–2.00)	1.08 (0.58–2.01)
**Constant**	2.79 (1.96–3.97)***	3.61 (2.02–6.43)***	0.98 (0.38–2.53)	0.69 (0.24–2.00)
**Likelihood**	–1263	–1231	–1132	–1116
**R-square**	0.01	0.03	0.11	0.12
**Goodness of fit**	0.151	0.593	0.938	0.661
**AIC**	2536	2481	2324	2299
**BIC**	2565	2533	2497	2489

Authors’ calculations based on Vietnam MICS 2020–2021. Model 1 adjusts for child gender and birth order. Model 2 adds child characteristics. Model 3 adds all covariates. Model 4 additionally includes interaction terms between child gender and birth order. svy was used to account for survey design. Ref. = reference group; OR = odds ratio; CI = confidence interval. *p < .05, **p < .01, ***p < .001.

When additional covariates were introduced in Models 2 and 3, the relationship between child gender and ECD remained non-significant, indicating that gender differences in overall developmental status were not evident among Vietnamese children aged 24–59 months after adjusting for child, maternal, and household characteristics. However, a different picture emerged once the interaction term between child gender and birth order was included in Model 4. The interaction term was jointly significant (Wald test, results not shown), suggesting that the association between gender and ECD varies by birth order. In other words, birth order plays a critical moderating role in shaping gender differences in ECD.

**Fig 1:**
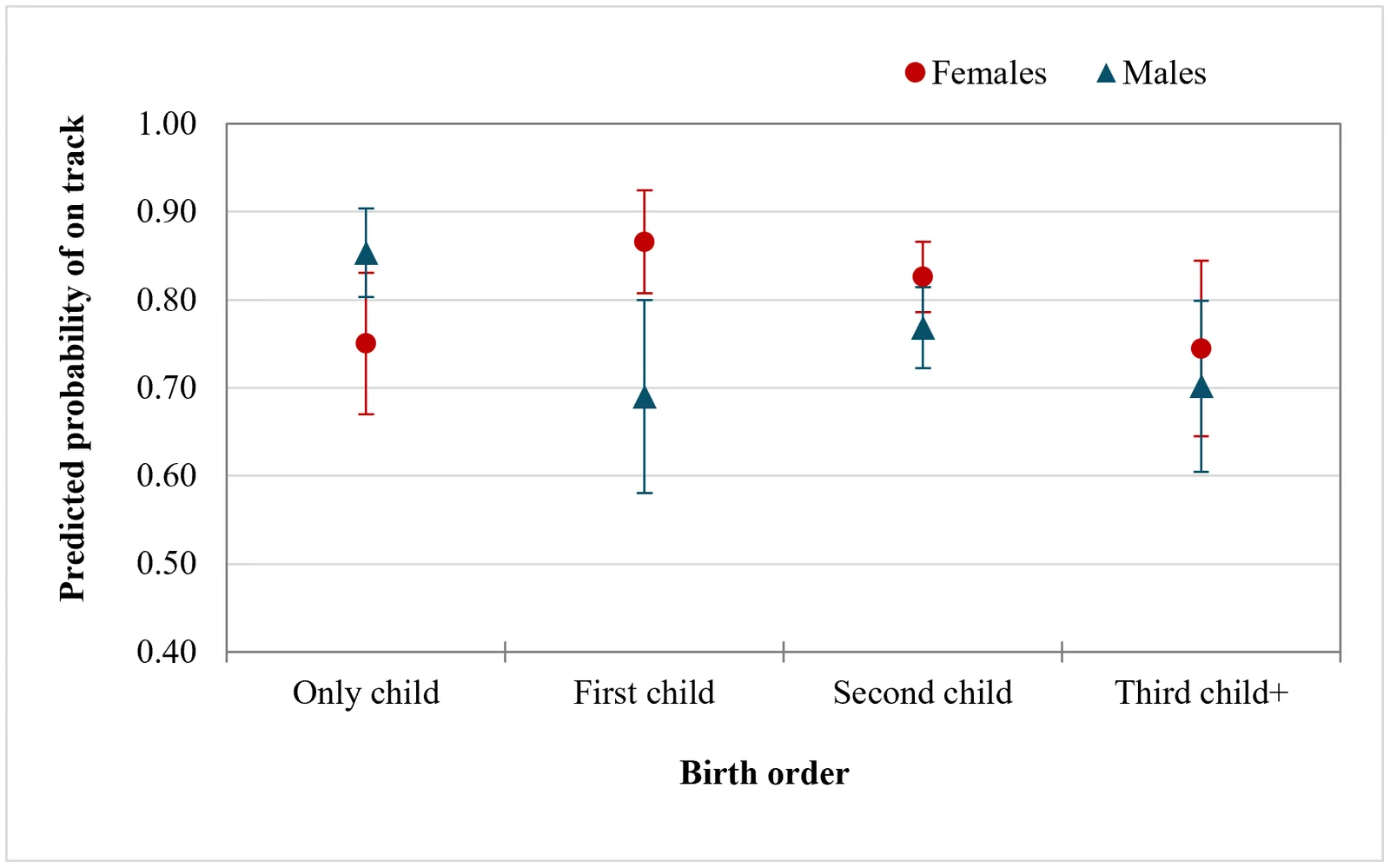
Average predicted probability of being on track by child gender and birth order (N = 2,354). Authors’ calculation based on Vietnam MICS 2020–2021. The estimates are based on Model 4, which controlled for all covariates. Ninety-five per cent confidence intervals are presented.

Fig 1 illustrates these interaction effects using average predicted probabilities of being on track. Firstborn daughters showed the most favourable development outcome, with an average predicted probability of 0.87 (95% CI: 0.81–0.92), significantly higher than that of sons at most birth orders, as indicated by non-overlapping CIs. Among daughters, the lowest probability was observed for third-born or higher (0.74; 95% CI: 0.64–0.84). Among sons, being the first child was associated with the lowest developmental probability (0.69; 95% CI: 0.58–0.80), while only sons had the highest probability (0.85; 95% CI: 0.80–0.90). However, the result for only children should be interpreted with caution due to greater uncertainty, as discussed further below.

### Home influences on early childhood development

Consistent with the descriptive results in Table 1, maternal education remained a strong predictor of ECD in the multivariate models. In Model 4, children of mothers with lower secondary education had 1.65 times higher odds of being on track in development (95% CI: 1.08–2.54). A similar advantage was observed for children of mothers with upper secondary education or higher (OR = 1.58; 95% CI: 1.01–2.47).

A supportive home environment was also strongly associated with better developmental outcomes among children aged 24–59 months. Children with 10 or more books had 2.40 times higher odds of being on track (95% CI: 1.34–4.29) compared with children with no books. Having 1–9 books increased the odds of being on track, but not significantly. Engagement in 5–6 stimulating activities in the previous three days – such as reading, storytelling, or playing – was associated with 1.48 times higher odds of being on track (95% CI: 1.09–2.02).

Furthermore, children who experienced psychological aggression without physical punishment had higher odds of being developmentally on track than children who received no discipline (OR = 2.05, 95% CI: 1.09–3.84). Finally, children from the richest households had 1.56 times the odds of being developmentally on track (95% CI: 1.04–2.33) compared with children from all other wealth groups.

## Discussion

This study, to our knowledge, is the first attempt to examine gender differences in ECD in Vietnam, using the most up-to-date Vietnam MICS 2020–2021. Despite Vietnam’s well-documented history of son preference, we found no overall gender difference in ECD among children aged 24–59 months, a pattern consistent with descriptive statistics in earlier MICS rounds from 2011 and 2014 [42,43]. The lack of negative ECD impacts for girls reveals a specific demographic pattern in Vietnam where discrimination is highly concentrated at the prenatal stage, pauses during early childhood, and then aggressively re-emerges later in the life course. Recent national research in Vietnam confirms that postnatal gendered disparities are highly age-dependent, with no significant gender differences in early education access among school-aged children [33], but significant emerging later in income, labour force participation, domestic work, and household leadership, as girls are increasingly socialised into and burdened by domestic and caregiving roles [44].

However, when birth order was introduced as a moderator, important gendered patterns emerged. The probability of being on track differed substantially across combinations of gender and birth order, indicating that gender inequality in ECD becomes visible only when examined through the lens of sibship structure. There are several possible reasons explaining why daughters – particularly firstborn daughters – exhibited developmental advantages, and why certain groups of sons faced relative disadvantages. The first plausible explanation lies in selection effects operating through Vietnam’s high prevalence of sex-selective abortion [25]. This selection started at the first birth because Vietnam’s SRB was already high at the first birth, while roughly normal for the second child, and highest at the third birth [19,37,45]. As a result, daughters – especially firstborn daughters – are likely born into families with weaker son preference, similar to what was concluded in a previous study in India [46].

In addition, while son preference persists, contemporary Vietnamese families increasingly aspire to a mixed-gender ideal: one boy and one girl [36,47]. Within this norm, firstborn daughters (“con gái đầu lòng”) are often valued because they move families closer to the ideal two-child composition. This mixed-gender preference may reduce gender discrimination for daughters in early childhood and potentially enhance the investment they receive.

Biological differences may also contribute to the observed patterns. Females possess a well-documented biological survival advantage, whereas male children are generally more vulnerable to early-life environmental shocks [27]. Evidence from settings experiencing severe food insecurity suggests that boys may be more susceptible to growth faltering and developmental delays than girls, as biological fragility interacts with nutritional deprivation and other environmental stressors [48]. However, these differences are context-specific and should not be interpreted as implying greater nutritional needs or preferential treatment for boys.

Moreover, behavioural factors such as temperament also play a role, with girls generally exhibiting higher levels of conscientiousness and self-regulation, which may facilitate smoother interactions with parents or caregivers, leading to more positive developmental stimulation [49]. These biological and behavioural factors may produce advantages for girls across most birth orders, independent of cultural norms.

A third mechanism relates to differences in parental investment by gender and birth order. Resource dilution theory posits that parental time, cognitive stimulation, and emotional resources decline as the number of children increases [9]. Our snapshot findings align with this theory: third-born and later-born children of both genders had lower developmental probabilities [50,51]. Only sons likely benefited from the greatest concentration of household resources, while firstborn sons experienced a significant developmental drop following the birth of a sibling as resources were divided. This pattern was not observed among daughters, suggesting that firstborn girls possibly maintain high developmental stability regardless of household expansion.

Firstborn daughters benefited from both being firstborn (higher resource investment) and from the selection effects discussed earlier. Conversely, firstborn sons experienced the lowest developmental outcomes, different from expectations derived from Vietnam’s patrilineal tradition [21]. Eldest sons (“cháu đích tôn”) hold the highest cultural significance, including ancestral worship duties and the expectation of co-residing with ageing parents [52]; however, this status may paradoxically lead to overprotective parenting.

Son preference in Vietnam potentially imposes a “cost” on boys, particularly firstborn sons. The extreme cultural significance placed on firstborn sons could lead to heightened parental anxiety and protective behaviours that limit their development, especially in a low-risk environment [53]. Evidence from urban Vietnam shows that maternal overprotection is common and associated with adolescent mental health problems [54]. International evidence further links overprotection to poorer psychosocial development [55], lower physical growth [56], and delayed motor and problem-solving development [56]. Meanwhile, daughters raised under more relaxed expectations could reach their full developmental potential.

Furthermore, the only-child category showed an apparent disadvantage for girls. In Vietnamese households with son preference, the only sons would benefit from being the firstborn, as discussed earlier, and being the preferred sex by culture, resulting in receiving more nurturing care and developmental resources than only daughters in families that continue to desire a son [12,19]. However, within the cross-sectional data of MICS6, this pattern likely reflects transitional fertility behaviour rather than stable gender inequality. Because the majority (90.7%) of mothers with only one child were under age 34, many only children are likely to be firstborns in families that will later have a second child, consistent with prevailing fertility patterns in Vietnam [57]. The uncertainty in measuring only children reinforces the need to interpret results with caution. As fertility continues to decline in Vietnam [57], monitoring gender differences among completed one-child families will become increasingly important, given parallels with China’s one-child era [20].

Beyond gender and birth order, our findings highlight several well-established determinants of ECD. Absence of disability [58], higher maternal education at the upper secondary level or higher [59,60], access to 10 or more children’s books [61,62], family engagement in multiple stimulating activities [63], and household wealth [58,62,64] were all positively associated with developmental outcomes. Variations in ECD across these contextual characteristics also reflect socioeconomic inequalities in child development, consistent with earlier studies in Vietnam [28,65,66]. These inequalities represent a major barrier to realising the WHO’s “Leave no child behind” goal in nurturing care [4], highlighting the need for interventions that address resource constraints, access to learning materials, and caregiver support across socioeconomically disadvantaged populations.

Our study found higher odds of being developmentally on track among children who experienced psychological aggression without physical punishment compared with those with no discipline at all. This finding should be interpreted with caution. One possible explanation is that, in Vietnam, psychologically aggressive disciplinary practices remain relatively common and may reflect greater parental involvement and interaction with children rather than a beneficial effect of psychological aggression itself. As the reference group comprised children who received no reported discipline, the observed association may capture differences in parental engagement rather than the effects of disciplinary practices [67]. However, substantial evidence has documented the adverse consequences of psychological aggression and other forms of violent discipline for children's well-being and development [33]. Given the cross-sectional nature of the data, the underlying mechanisms cannot be determined, and further research is needed to better understand this relationship.

Finally, this study has several limitations. A primary limitation is the cross-sectional design of MICS6, which does not allow for the establishment of a definitive causal relationship [68] and captures early childhood development [only at a single point in time, while child development changes over the life course [3]. The examination of ongoing families in cross-sectional data may be subject to birth-order misclassification, particularly among only children. In fact, restricting to completed fertility, such as using mothers aged 35 or 40 and above, may reduce this misclassification but would introduce substantial selection concerns and reduce statistical power due to a smaller sample size. Although the statistical results from the restricted model excluding all only children were consistent with those from the main results (S3 Table), suggesting that the observed female advantage is not driven by the inclusion of potentially misclassified cases, longitudinal data would be needed to definitively assess birth-order effects after family completion.

Additionally, the inclusion of 183 households with multiple siblings in our study may introduce within-household clustering. However, we used survey-adjusted models (svy in Stata), accounting for sampling weights, stratification, and primary sampling units. This approach adjusts standard errors for the complex survey design and potential non-independence among observations. The restricted model, excluding households with multiple siblings, yielded findings consistent with our main results on the advantage of girls (S4 Table).

Factors such as nutritional status [62,69], breastfeeding, immunisation, children’s temperament, father’s characteristics, and the healthcare system [8], which are likely to affect child development, were not included in this study due to constraints of secondary data. The preschool attendance variable, although available, was excluded due to significant age-related missingness, particularly among two-year-olds. Several measures – including functional difficulty, discipline, engagement, and number of books – capture quantity rather than quality, potentially limiting their interpretive precision. Future studies should use primary data to address these limitations and provide more comprehensive findings.

## Conclusions

Using nationally representative MICS 2020–2021 data, this study provides the first detailed analysis of gender differences in early childhood development in Vietnam within a gender and birth-order framework. Despite Vietnam’s longstanding history of son preference, the study reveals no overall gender gap in developmental outcomes. However, gender differences emerged once birth order was considered: firstborn daughters showed the highest developmental probabilities, whereas firstborn sons exhibited the lowest. In addition, maternal education, home learning resources, caregiver engagement, positive discipline, and household wealth were strongly associated with better development, underscoring persistent socioeconomic disparities. While these findings highlight how gender inequality in Vietnam manifests not broadly but through specific family contexts, limitations of cross-sectional data and unmeasured factors such as nutrition and parenting quality call for caution. Strengthening nurturing care environments and supporting equitable caregiving across all birth orders may help ensure that every Vietnamese child has an equal opportunity to thrive.

## Supporting information

S1 Table Probit Regression Model, N = 2354. (DOCX)

S2 Table Logistic regression results without survey weights, N = 2354. (DOCX)

S3 Table Logistic regression results excluding only children, N = 1893. (DOCX)

S4 Table Logistic regression results excluding households with multiple siblings. (DOCX)
