# Supplementary material for: Early childhood development under son preference in Vietnam: The role of child gender and birth order
Source: PLOS Glob Public Health. 2026 Aug 27;6(8):e0006983. doi: 10.1371/journal.pgph.0006983 (PMC13521366; doi:10.1371/journal.pgph.0006983)
Supplement: Supplementary file 1 [file pgph.0006983.s001.docx]

**S1 Table: Probit Regression Model, N = 2354**

| **Variables** | **Coeff (95% CI)** |
| --- | --- |
| Child gender (Ref. Male) |  |
| Female | 0.70 (0.25; 1.15)** |
| Birth order (Ref. First child) |  |
| Only child | 0.65 (0.25; 1.05)** |
| Second child | 0.28 (-0.13; 0.69) |
| Third child+ | 0.08 (-0.40; 0.55) |
| Interaction: Child gender × Birth order |  |
| Female × Only child | -1.12 (-1.67; -0.57)*** |
| Female × Second child | -0.45 (-0.97; 0.08) |
| Female × Third child+ | -0.56 (-1.16; 0.04) |
| Gender composition of siblings (Ref. Having an older brother) |  |
| No older brother | 0.03 (-0.17; 0.24) |
| Child age (Ref. 2 years) |  |
| 3 years | -0.16 (-0.37; 0.04) |
| 4 years | -0.36 (-0.57; -0.14)** |
| Functional difficulty (Ref. No) |  |
| Yes | -1.77 (-2.54; -1.01)*** |
| Mother’s age at birth (Ref. 12–24) |  |
| 25–29 | 0.11 (-0.10; 0.32) |
| 30–44 | 0.01 (-0.23; 0.26) |
| Mother’s education (Ref. Primary and below) |  |
| Lower secondary | 0.30 (0.05; 0.56)* |
| Upper secondary+ | 0.28 (0.02; 0.55)* |
| Marital status (Ref. Currently married) |  |
| Formerly married | -0.23 (-0.66; 0.21) |
| Never married | -1.38 (-2.54; -0.21)* |
| Providing books (Ref. None) |  |
| 1 to 9 books | 0.20 (-0.02; 0.41) |
| 10+ books | 0.48 (0.18; 0.77)** |
| Child’s engagement (Ref: 0–4 activities) |  |
| 5–6 activities | 0.22 (0.05; 0.40)* |
| Child’s discipline (Ref. No discipline) |  |
| Non-violent discipline | 0.15 (-0.18; 0.48) |
| Psychological aggression | 0.41 (0.04; 0.77)* |
| Physical punishment | 0.17 (-0.16; 0.49) |
| Household wealth (Ref. Others) |  |
| Rich/ richest | 0.24 (0.01; 0.46)* |
| Household size (Ref. 2–4 members) |  |
| 5–17 members | 0.14 (-0.03; 0.31) |
| Ethnicity (Ref. Minorities) |  |
| Kinh and Hoa | 0.18 (-0.04; 0.40) |
| Residence (Ref. Rural) |  |
| Urban | 0.07 (-0.14; 0.28) |
| Region (Ref. Red River) |  |
| Northern Midlands | -0.18 (-0.47; 0.11) |
| North Central | -0.28 (-0.58; 0.01) |
| Central Highlands | -0.12 (-0.47; 0.23) |
| South East | -0.34 (-0.61; -0.08)* |
| Mekong River | 0.05 (-0.29; 0.39) |
| **Constant** | -0.22 (-0.83; 0.40) |

Authors’ calculation based on Vietnam MICS 2020 – 2021. The model included children, the mother, the home environment, the household characteristics, and the interaction term between child gender and birth order. svy was used to account for survey design. Ref: reference group. Coeff: Coefficient. CI: Confidence Interval. **p* < .05, ** *p* < .01, ****p* < .001.
