# Supplementary material for: Early childhood development under son preference in Vietnam: The role of child gender and birth order
Source: PLOS Glob Public Health. 2026 Aug 27;6(8):e0006983. doi: 10.1371/journal.pgph.0006983 (PMC13521366; doi:10.1371/journal.pgph.0006983)
Supplement: Supplementary file 2 [file pgph.0006983.s002.docx]

**S2 Table: Logistic regression results without survey weights, N=2354**

|  | **Likelihood of being on track for development** | |
| --- | --- | --- |
| **Variables** | **Weighted Model** | **Unweighted Model** |
|  | OR (95% CI) | OR (95% CI) |
| Child gender (Ref. Male) |  |  |
| Female | 3.34 (1.46–7.63)** | 1.40 (0.89–2.20) |
| Birth order (Ref. First child) |  |  |
| Only child | 2.98 (1.47–6.04)** | 1.51 (0.95–2.41) |
| Second child | 1.58 (0.77–3.23) | 0.99 (0.66–1.49) |
| Third child+ | 1.10 (0.47–2.43) | 1.08 (0.65–1.81) |
| Interaction: Child gender × Birth order | |  |
| Female × Only child | 0.14 (0.05–0.38)*** | 0.53 (0.27–1.02) |
| Female × Second child | 0.45 (0.17–1.16) | 1.12 (0.66–1.93) |
| Female × Third child+ | 0.38 (0.13–1.11) | 0.70 (0.37–1.34) |
| Gender composition of siblings (Ref. Having an older brother) | |  |
| No older brother | 1.04 (0.73–1.49) | 1.11 (0.86–1.43) |
| Child age (Ref. 2 years) |  |  |
| 3 years | 0.73 (0.51–1.05) | 0.66 (0.51–0.85)** |
| 4 years | 0.52 (0.36–0.76)** | 0.44 (0.34–0.57)*** |
| Functional difficulty (Ref. No) |  |  |
| Yes | 0.05 (0.01–0.20)*** | 0.03 (0.01–0.12)*** |
| Mother’s age at birth (Ref. 12–24) |  |  |
| 25–29 | 1.20 (0.83–1.73) | 1.37 (1.06–1.78)* |
| 30–44 | 1.03 (0.67–1.57) | 1.27 (0.97–1.66) |
| Mother’s education (Ref. Primary and below) | |  |
| Lower secondary | 1.65 (1.08–2.54)* | 1.63 (1.24–2.13)*** |
| Upper secondary+ | 1.58 (1.01–2.47)* | 1.69 (1.26–2.28)** |
| Marital status (Ref. Currently married) | |  |
| Formerly married | 0.68 (0.31–1.46) | 0.64 (0.35–1.18) |
| Never married | 0.10 (0.01–0.70)* | 0.33 (0.07–1.63) |
| Providing books (Ref. None) |  |  |
| 1 to 9 books | 1.42 (0.97–2.09) | 1.51 (1.11–2.07)* |
| 10+ books | 2.40 (1.34–4.29)** | 3.28 (1.89–5.69)*** |
| Child’s engagement (Ref: 0–4 activities) | |  |
| 5–6 activities | 1.48 (1.09–2.02)* | 1.41 (1.10–1.81)** |
| Child’s discipline (Ref. No discipline) |  |  |
| Non-violent discipline | 1.32 (0.75–2.31) | 1.95 (1.31–2.92)** |
| Psychological aggression | 2.05 (1.09–3.84)* | 2.42 (1.62–3.63)*** |
| Physical punishment | 1.38 (0.79–2.39) | 1.96 (1.37–2.82)*** |
| Household wealth (Ref. Others) |  |  |
| Rich/ richest | 1.56 (1.04–2.33)* | 1.26 (0.90–1.78) |
| Household size (Ref. 2–4 members) |  |  |
| 5–17 members | 1.28 (0.95–1.98) | 1.17 (0.94–1.45) |
| Ethnicity (Ref. Minorities) |  |  |
| Kinh and Hoa | 1.37 (0.94–1.98) | 1.34 (1.01–1.79)* |
| Residence (Ref. Rural) |  |  |
| Urban | 1.12 (0.77–1.63) | 1.27 (0.94–1.73) |
| Region (Ref. Red River) |  |  |
| Northern Midlands | 0.76 (0.45–1.27) | 0.64 (0.41–0.99)* |
| North Central | 0.62 (0.36–1.05) | 0.70 (0.45–1.09) |
| Central Highlands | 0.82 (0.45–1.51) | 0.66 (0.42–1.04) |
| South East | 0.55 (0.34–0.89)* | 0.53 (0.34–0.83)** |
| Mekong River | 1.08 (0.58–2.01) | 1.04 (0.64–1.67) |
| **Constant** | 0.69 (0.24–2.00) | 0.79 (0.39–1.62) |

Authors’ calculation based on Vietnam MICS 2020 – 2021. The model included children, the mother, the home environment, the household characteristics, and the interaction term between child gender and birth order. Ref: reference group. OR: Odds Ratio. CI: Confidence Interval. **p* < .05, ** *p* < .01, ****p* < .001.
