# Supplementary material for: Early childhood development under son preference in Vietnam: The role of child gender and birth order
Source: PLOS Glob Public Health. 2026 Aug 27;6(8):e0006983. doi: 10.1371/journal.pgph.0006983 (PMC13521366; doi:10.1371/journal.pgph.0006983)
Supplement: Supplementary file 3 [file pgph.0006983.s003.docx]

**S3 Table: Logistic regression results excluding only children, N=1893**

|  | **Likelihood of being on track for development** |
| --- | --- |
| **Variables** | **Model excluding only children** |
|  | OR (95% CI) |
| Child’s gender (Ref. Male) |  |
| Female | 3.30 (1.46–7.46)** |
| Birth order (Ref. First child) |  |
| Second child | 1.57 (0.76–3.25) |
| Third child+ | 1.02 (0.44–2.36) |
| Interaction: Child’s gender × Birth order |  |
| Female × Second child | 0.46 (0.18–1.16) |
| Female × Third child+ | 0.38 (0.13–1.09) |
| Gender composition of siblings (Ref. Having an older brother) | |
| No older brother | 1.04 (0.73–1.49) |
| Child’s age (Ref. 2 years) |  |
| 3 years | 0.68 (0.45–1.02) |
| 4 years | 0.49 (0.32–0.75)** |
| Functional difficulty (Ref. No) |  |
| Yes | 0.05 (0.01–0.21)*** |
| Mother’s age at birth (Ref. 12–24) |  |
| 25–29 | 1.08 (0.70–1.65) |
| 30–44 | 1.05 (0.63–1.73) |
| Mother’s education (Ref. Primary and below) | |
| Lower secondary | 1.71 (1.07–2.73)* |
| Upper secondary+ | 1.58 (0.95–2.63) |
| Marital status (Ref. Currently married) |  |
| Formerly married | 0.69 (0.23–2.06) |
| Never married | 0.25 (0.01–5.37) |
| Providing books (Ref. None) |  |
| 1–9 books | 1.52 (0.98–2.35) |
| 10+ books | 2.42 (1.22–4.78)* |
| Child’s engagement (Ref: 0–4 activities) |  |
| 5–6 activities | 1.29 (0.91–1.82) |
| Child’s discipline (Ref. No discipline) |  |
| Non-violent discipline | 1.11 (0.55–2.23) |
| Psychological aggression | 1.81 (0.88–3.72) |
| Physical punishment | 1.15 (0.61–2.20) |
| Household wealth (Ref. Others) |  |
| Rich/richest | 1.54 (0.98–2.41) |
| Household size (Ref. 2–4 members) |  |
| 5–17 members | 1.29 (0.92–1.82) |
| Ethnicity (Ref. Minorities) |  |
| Kinh and Hoa | 1.44 (0.95–2.19) |
| Residence (Ref. Rural) |  |
| Urban | 1.05 (0.68–1.63) |
| Region (Ref. Red River) |  |
| Northern Midlands | 0.75 (0.43–1.31) |
| North Central | 0.65 (0.35–1.20) |
| Central Highlands | 0.83 (0.44–1.59) |
| South East | 0.52 (0.30–0.89)* |
| Mekong River | 1.05 (0.52–2.11) |
| **Constant** | 0.89 (0.29–2.69) |

Authors’ calculation based on Vietnam MICS 2020 – 2021. svy was used to account for survey design. Ref: reference group. OR: odds ratio. CI: Confidence Interval. **p* < .05, ** *p* < .01, ****p* < .001.
