# Supplementary material for: Early childhood development under son preference in Vietnam: The role of child gender and birth order
Source: PLOS Glob Public Health. 2026 Aug 27;6(8):e0006983. doi: 10.1371/journal.pgph.0006983 (PMC13521366; doi:10.1371/journal.pgph.0006983)
Supplement: Supplementary file 4 [file pgph.0006983.s004.docx]

**S4 Table: Logistic regression results excluding households with multiple siblings**

|  | **Likelihood of being on track for development** | |
| --- | --- | --- |
| **Variables** | **Model 1**  (all households) | **Model 2**  (excluding households with multiple siblings) |
|  | OR (95% CI) | OR (95% CI) |
| Child’s gender (Ref. Male) |  |  |
| Female | 3.34 (1.46–7.63)** | 2.98 (1.05–8.42)* |
| Birth order (Ref. First child) |  |  |
| Only child | 2.98 (1.47–6.04)** | 3.38 (1.55–7.36)** |
| Second child | 1.58 (0.77–3.23) | 1.77 (0.79–3.96) |
| Third child+ | 1.10 (0.47–2.43) | 1.34 (0.55–3.27) |
| Interaction: Child’s gender × Birth order |  |  |
| Female × Only child | 0.14 (0.05–0.38)*** | 0.16 (0.05–0.50)** |
| Female × Second child | 0.45 (0.17–1.16) | 0.50 (0.16–1.56) |
| Female × Third child+ | 0.38 (0.13–1.11) | 0.39 (0.11–1.34) |
| Gender composition of siblings (Ref. Having an older brother) | |  |
| No older brother | 1.04 (0.73–1.49) | 0.99 (0.67–1.45) |
| Child’s age (Ref. 2 years) |  |  |
| 3 years | 0.73 (0.51–1.05) | 0.80 (0.54–1.17) |
| 4 years | 0.52 (0.36–0.76)** | 0.51 (0.35–0.77)** |
| Functional difficulty (Ref. No) |  |  |
| Yes | 0.05 (0.01–0.20)*** | 0.04 (0.01–0.17)*** |
| Mother’s age at birth (Ref. 12–24) |  |  |
| 25–29 | 1.20 (0.83–1.73) | 1.12 (0.76–1.65) |
| 30–44 | 1.03 (0.67–1.57) | 0.87 (0.55–1.36) |
| Mother’s education (Ref. Primary and below) | |  |
| Lower secondary | 1.65 (1.08–2.54)* | 1.40 (0.88–2.24) |
| Upper secondary+ | 1.58 (1.01–2.47)* | 1.31 (0.82–2.10) |
| Marital status (Ref. Currently married) |  |  |
| Formerly married | 0.68 (0.31–1.46) | 0.64 (0.30–1.39) |
| Never married | 0.10 (0.01–0.70)* | 0.10 (0.02–0.69)* |
| Providing books (Ref. None) |  |  |
| 1–9 books | 1.42 (0.97–2.09) | 1.43 (0.95–2.15) |
| 10+ books | 2.40 (1.34–4.29)** | 2.89 (1.56–5.38)** |
| Child’s engagement (Ref: 0–4 activities) |  |  |
| 5–6 activities | 1.48 (1.09–2.02)* | 1.39 (1.00–1.92) |
| Child’s discipline (Ref. No discipline) |  |  |
| Non-violent discipline | 1.32 (0.75–2.31) | 1.15 (0.65–2.03) |
| Psychological aggression | 2.05 (1.09–3.84)* | 2.01 (1.06–3.80)* |
| Physical punishment | 1.38 (0.79–2.39) | 1.25 (0.70–2.24) |
| Household wealth (Ref. Others) |  |  |
| Rich/richest | 1.56 (1.04–2.33)* | 1.63 (1.08–2.46)* |
| Household size (Ref. 2–4 members) |  |  |
| 5–17 members | 1.28 (0.95–1.98) | 1.32 (0.95–1.82) |
| Ethnicity (Ref. Minorities) |  |  |
| Kinh and Hoa | 1.37 (0.94–1.98) | 1.36 (0.91–2.05) |
| Residence (Ref. Rural) |  |  |
| Urban | 1.12 (0.77–1.63) | 1.17 (0.79–1.74) |
| Region (Ref. Red River) |  |  |
| Northern Midlands | 0.76 (0.45–1.27) | 0.77 (0.42–1.39) |
| North Central | 0.62 (0.36–1.05) | 0.66 (0.38–1.14) |
| Central Highlands | 0.82 (0.45–1.51) | 0.86 (0.47–1.59) |
| South East | 0.55 (0.34–0.89)* | 0.55 (0.33–0.91)* |
| Mekong River | 1.08 (0.58–2.01) | 1.14 (0.58–2.25) |
| **Constant** | 0.69 (0.24–2.00) | 0.81 (0.24–2.71) |
| **Number of observations** | 2354 | 1984 |

Authors’ calculation based on Vietnam MICS 2020 – 2021. Svy was used to account for survey design. Ref: reference group. OR: odds ratio. CI: Confidence Interval. **p* < .05, ** *p* < .01, ****p* < .001
